# Supplementary material for: Diverse Effects of Cilostazol on Proprotein Convertase Subtilisin/Kexin Type 9 between Obesity and Non-Obesity
Source: Int J Mol Sci. 2022 Aug 29;23(17):9768. doi: 10.3390/ijms23179768 (PMC9456424; doi:10.3390/ijms23179768)
Supplement: Supplementary file 1 [file ijms-23-09768-s001.zip › ijms-1873313-supplementary/Table S1-3.pdf]

Table S1. Sequences of the primers for real-time polymerase chain reaction (RT-PCR)

Gene	Forward	Reverse
hPPARγ	CAGGCCGAGAAGGAGAAGCT	CAGGCCGAGAAGGAGAAGCT
mPPARγ	ATGTCTCACAATGCCATCAGGTT	GCTCGCAGATCAGCAGACTCT
hPCSK9	AGTTGCCCCATGTCGACTAC	GAGATACACCTCCACCAGGC
mPCSK9	TATGAAGAGCTGATGCTCGC	CACAATGTAGGTTCCTGGCA
hLDLR	GACGTGGCGTGAACATCTG	CTGGCAGGCAATGCTTTGG
mLDLR	GTATGAGGTTCCTGTCCATC	CCTCTGTGGTCTTCTGGTAG
hAdiponectin	AGGAAACCACGACTCAAG	ACCGATGTCTCCCTTAGG
hAdipoR1	CTATCGCTGAGGGCTTTG	AATCTGATGAGACTGGAAC
hAdipoR2	GCAGCCATTATAGTCTCC	AGCATCAACCAGCCTATC
hGADPH	AAGGTGAAGGTCGGAGTCAAC	TGGCAACAATATCCACTTTACC
mGADPH	ACCCAGAAGACTGTGGATGG	ACACATTGGGGGTAGGAACA

AdipoR1: adiponectin receptor 1; AdipoR2: adiponectin receptor 2; CAT: catalase; GAPDH: glyceraldehyde-3-phosphate dehydrogenase; h: homo sapiens; LDLR: low-density lipoprotein receptor; m: mus musculus; PCSK9: proprotein convertase subtilisin/kexin type 9; PPARγ: peroxisome proliferator-activated receptorγ.

Table S2. Sequences of the primers for promoter constructs

Gene	Forward	Reverse
hPPARγ	CACGCTCGAGTTTGGATAGCA GTAAC	ACGTAAGCTTTAGGGTTCTATGCT GA
hPCSK9	AGGGTACCCTGGACATCAAA AGCAAGC	AGCTCGAGCGCAGCGGTGGAAG GTG
hPCSK9-PPREmut	TGCAGGGTGCATAACAAGTTG TTCATCGAGGGGGTCCAGGC	GCCTGGACCCCCTCGATGAACAA CTTGTTATGCACCCTGCA
hPCSK9-SREmut	ATGGGGCTCTGAGATCCGTGT CTGCGCGCCCCA	TGGGGCGCGCAGACACGGATCT CAGAGCCCCAT
hLDLR	GCGGTACCCCTTTTGAGGCAG AGAGGACA	ACCTCGAGGGGCTCCCTCTCAAC CTATTC
hLDLR-SREmut	TGAAGACATTTGAAAATCACG GCACTGCAAACTCC	GGGGAGGAGTTTGCAGTGCCGT GATTTTCAAATGT

PPRE: peroxisome proliferator response element; SRE: sterol regulatory element

Table S3. Sequences of the primers for chromatin immunoprecipitation (ChIP)

Gene	Forward	Reverse
hPCSK9-PPREmut	ACGTCTTTGCAAACTTAAAAC CTG	GTTTCCTGGGTCCACCTTGT
hPCSK9-SPREmut	TTCCCTCTGCGCGTAATCTG	CAGACCCTGAACTGAACGGC
hLDLR-SREmut	TCGAAGGACTGGAGTGGGAA	GACCTGCTGTGTCCTAGCTG
